# Antibodies toward infliximab are associated with low infliximab concentration at treatment initiation and poor infliximab maintenance in rheumatic diseases

**DOI:** 10.1186/ar3386

**Published:** 2011-06-27

**Authors:** Emilie Ducourau, Denis Mulleman, Gilles Paintaud, Delphine Chu Miow Lin, Francine Lauféron, David Ternant, Hervé Watier, Philippe Goupille

**Affiliations:** 1 Université François Rabelais de Tours, Centre National de la Recherche Scientifique UMR 6239 GICC (Génétique Immunothérapie Chimie et Cancer), 3 rue des Tanneurs, F-37041 Tours Cedex 1, France; 2 Service de Rhumatologie, Centre Hospitalier Régional et Universitaire de Tours, avenue de la République, F-37044 Tours Cedex 9, France; 3 Laboratoire de Pharmacologie-Toxicologie, Centre Hospitalier Régional et Universitaire de Tours, 2 boulevard Tonnellé, F-37044 Tours Cedex 9, France; 4 Laboratoire d'Immunologie, Centre Hospitalier Régional et Universitaire de Tours, 2 boulevard Tonnellé, F-37044 Tours Cedex 9, France

## Abstract

**Introduction:**

A proportion of patients receiving infliximab have antibodies toward infliximab (ATI), which are associated with increased risk of infusion reaction and reduced response to treatment. We studied the association of infliximab concentration at treatment initiation and development of ATI as well as the association of the presence of ATI and maintenance of infliximab.

**Methods:**

All patients with rheumatoid arthritis (RA) or spondyloarthritis (SpA) receiving infliximab beginning in December 2005 were retrospectively followed until January 2009 or until infliximab discontinuation. Trough serum infliximab and ATI concentrations were measured at each visit. The patients were separated into two groups: ATI_pos _if ATI were detected at least once during the follow-up period and ATI_neg _otherwise. Repeated measures analysis of variance was used to study the association of infliximab concentration at treatment initiation and the development of ATI. Maintenance of infliximab in the two groups was studied by using Kaplan-Meier curves.

**Results:**

We included 108 patients: 17 with RA and 91 with SpA. ATI were detected in 21 patients (19%). The median time to ATI detection after initiation of infliximab was 3.7 months (1.7 to 26.0 months). For both RA and SpA patients, trough infliximab concentration during the initiation period was significantly lower for ATI_pos _than ATI_neg _patients. RA patients showed maintenance of infliximab at a median of 19.5 months (5.0 to 31.0 months) and 12.0 months (2.0 to 24.0 months) for ATI_neg _and ATI_pos _groups, respectively (*P *= 0.08). SpA patients showed infliximab maintenance at a median of 16.0 months (3.0 to 34.0 months) and 9.5 months (3.0 to 39.0 months) for ATI_neg _and ATI_pos _groups, respectively (*P *= 0.20). Among SpA patients, those who were being treated concomitantly with methotrexate had a lower risk of developing ATI than patients not taking methotrexate (0 of 14 patients (0%) vs. 25 of 77 patients (32%); *P *= 0.03).

**Conclusions:**

High concentrations of infliximab during treatment initiation reduce the development of ATI, and the absence of ATI may be associated with prolonged maintenance of infliximab. Thus, trough serum infliximab concentration should be monitored early in patients with rheumatic diseases.

## Introduction

Infliximab, a chimeric mAb targeting TNFα, is used to treat rheumatoid arthritis (RA), spondyloarthritis (SpA) and inflammatory bowel diseases. Its efficacy and safety have been evaluated in selected patients in pivotal clinical trials [1-3], but predictive factors regarding its maintenance in the postmarketing clinical setting have not been reported. Because of its immunogenicity, infliximab is responsible for the development of antibodies toward infliximab (ATI), which is associated with increased risk of treatment failure. In RA, the development of ATI is inversely proportional to the dosage of infliximab [1], and low trough serum infliximab concentration 1.5 months after initiation is associated with the development of ATI [4]. Moreover, ATI are associated with increased risk of infusion reactions and decreased response to infliximab [4,5]. Trough serum infliximab concentration has been measured in SpA in only two studies. De Vries *et al*. [6] found that treatment failure is associated with low serum concentration and that the development of ATI is associated with undetectable trough infliximab concentration, reduced response to treatment and increased risk of infusion reactions. In contrast, Krzysiek *et al*. [7] did not find any association of trough infliximab concentration and response to treatment. Therefore, the relationships among infliximab concentration, development of ATI and response to treatment are less clear in SpA than in RA. Moreover, no study has focused on the temporal relationship between trough infliximab concentration and development of ATI.

We studied the association of trough serum infliximab concentration measured at treatment initiation and the development of ATI in a retrospective cohort with inflammatory rheumatic diseases. We also studied the association of ATI, infusion-related reactions and maintenance of infliximab.

## Materials and methods

### Patients

Patients with RA and patients with SpA whose infliximab treatment was started between December 2005 and January 2009 or until infliximab discontinuation were retrospectively included. Demographic characteristics, mean disease duration and concomitant treatment with methotrexate (MTX) or prednisone were recorded before infliximab initiation. RA patients received 3 mg/kg infliximab intravenously (rounded in the 100-mg vial) at weeks 0, 2, 6 and 14 and every 8 weeks thereafter, and SpA patients received 5 mg/kg infliximab (rounded in the 100-mg vial) at weeks 0, 2, 6 and 12 and every 6 weeks thereafter. The time and circumstances of discontinuation were recorded. The treatment protocol was in accordance with the guidelines of the French Society of Rheumatology for the use of infliximab [8,9]. Ethical approval and informed consent were not sought in this retrospective analysis of routine patients, which is in accordance with institutional guidelines.

### Clinical measurements

Before proceeding with the first infusion (baseline) and at each subsequent infusion, patients were asked about any adverse events since the previous visit and underwent a physical examination and urine analysis to rule out any concomitant infection. At each visit, the Disease Activity Score in 28 joints was measured for RA patients and the Bath Ankylosing Spondylitis Disease Activity Index (BASDAI) was used to assess disease activity in SpA patients. Blood samples were obtained 48 hours before each infusion for routine measurement of erythrocyte sedimentation rate (ESR) and C-reactive protein level (CRP).

### Infliximab serum and ATI concentrations

Serum samples were obtained just before each infusion for infliximab concentration measurement and ATI detection within the framework of routine therapeutic drug monitoring. The samples were not drawn specifically for this study, which was performed retrospectively. Infliximab concentrations were measured by ELISA as described previously [10]. Serum concentration of ATI was measured by double-antigen ELISA on the basis of capture by infliximab-coated microplates and detection by peroxidase-conjugated infliximab [11]. This assay was standardised by the use of a mouse mAb against human immunoglobulin G. The positive threshold of detection was 0.07 mg/L. Because of the interference of circulating infliximab, only sera with infliximab concentration < 2 mg/L were tested. Patients were separated into two groups: ATI_pos _if ATI were detected at least once during follow-up and ATI_neg _otherwise.

### Dose adjustment

Infliximab dose could be adapted after the fourth infusion. The decision to increase, decrease or discontinue infliximab took into account the disease activity assessment on the one hand and infliximab trough concentration on the other hand. The principle underlying this drug monitoring procedure was previously described [12].

### Statistical analysis

Baseline characteristics of ATI_pos _and ATI_neg _groups were compared by using Student's *t*-test or a χ^2 ^test. Repeated measures analysis of variance was used to study the association of infliximab concentration during treatment initiation and the development of ATI. Maintenance of infliximab was studied by using Kaplan-Meier curves, and groups were compared by using a logrank test. Statistical analysis involved use of R version 2.7.2 software [13]. *P *< 0.05 was considered statistically significant. Results are presented as medians (full ranges) unless otherwise stated.

## Results

### Baseline characteristics of patients

**Figure 1:**
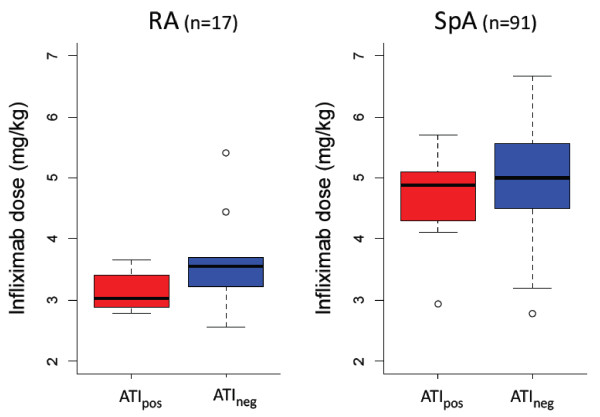
**Initial infliximab dose for patients positive and negative for antibodies toward infliximab (ATI_pos _and ATI_neg_, respectively)**. Box plots show the medians and interquartile ranges, and the whisker plots represent the 95th percentiles. Two patients in the RA subgroup (open circles) received 5 mg/kg infliximab at initiation because they were initially suspected of having psoriatic arthritis. During follow-up, anticitrullinated protein antibodies were detected in both patients, which explains their placement in the RA subgroup. Two patients with SpA (open circles) received 3 mg/kg infliximab at initiation because they had a peripheral form of the disease and were therefore misclassified as having RA. ATI: antibodies toward infliximab; ATI_pos_: ATI detected at least once during follow-up; ATI_neg_: ATI not detected; RA: rheumatoid arthritis; SpA: spondyloarthritis.

We included 108 patients: 17 with RA and 91 with SpA. ATI, which were undetectable in all patients before the initiation of infliximab therapy, were detected in 21 patients (7 with RA and 14 with SpA) during follow-up. The proportion of ATI_pos _patients was higher among those with RA than in patients with SpA (41% vs. 15%, respectively; *P *= 0.03). The baseline characteristics of the patients are given in Table 1. The ATI_pos _and ATI_neg _patients did not differ with regard to age, body mass index, concomitant treatment with prednisone or ESR or CRP level. For SpA patients, disease duration was longer, but not significantly so, for the ATI_pos _group than for the ATI_neg _group. Median time of ATI detection after initiation was 3.7 months (1.7 to 26.0 months). For RA patients, the infliximab dose was lower, but not significantly so, for the ATI_pos _patients than for the ATI_neg _patients (Table 1 and Figure 1). For SpA patients, concomitant MTX treatment was lower for ATI_pos _than for ATI_neg _patients (0 (0%) of 14 vs. 25 (32%) of 77, respectively; *P *= 0.03) (Tables 1 and 2).

**Table 1:** Baseline characteristics of the patients^a^

	RA (*n *= 17)			SpA (*n *= 91)		
	
Characteristics	ATI_pos _(*n *= 7)	ATI_neg _(*n *= 10)	*P *value	ATI_pos _(*n *= 14)	ATI_neg _(*n *= 77)	*P *value
Age, years	49 (28 to 65)	47 (36 to 64)	0.7	47 (36 to 73)	44 (14 to 76)	0.2
Body mass index, kg/m^2^	25.9 (20.2 to 34.8)	23.5 (16.4 to 34.6)	0.5	24.8 (17.5 to 32.8)	26.1 (15.8 to 45.8)	0.1
Disease duration, years	6.0 (1 to 12)	10 (2 to 30)	0.07	8.9 (0 to 24)	5 (0 to 24)	0.3
Infliximab dose, mg/kg	3.0 (2.8 to 3.7)	3.6 (2.6 to 5.4)	0.09	4.9 (2.9 to 5.7)	5.0 (2.8 to 6.7)	0.4
Concomitant treatments						
MTX, *n *(%)	3 (43)	6 (60)	0.8	0 (0)	25 (32)	0.03
Prednisone, *n *(%)	4 (57)	8 (80)	0.6	2 (14)	12 (16)	0.8
ESR, mm/hour	14 (7 to 58)	24 (2 to 114)	0.5	8 (2 to 54)	10 (1 to 89)	0.6
CRP, mg/L	10 (3 to 81)	16 (3 to 124)	0.9	3 (1 to 72)	5 (1 to 74)	0.9

^a^ATI: antibodies toward infliximab; ATI_pos_: ATI detected at least once during follow-up; ATI_neg_: ATI not detected; RA: rheumatoid arthritis; SpA: spondyloarthritis; MTX: methotrexate; ESR: erythrocyte sedimentation rate; CRP: C-reactive protein. Results are given as medians (ranges) unless otherwise indicated.

**Table 2:** Development of ATI by MTX treatment in RA and SpA patients^a^

	RA (*n *= 17)			SpA (*n *= 91)		
	
MTX treatment	ATI_pos_	ATI_neg_	*P *value	ATI_pos_	ATI_neg_	*P *value
MTX+	3	6		0	25	
MTX-	4	4	0.8	14	52	0.03

^a^ATI: antibodies toward infliximab; ATI_pos_: ATI detected at least once during follow-up; ATI_neg_: ATI not detected; RA: rheumatoid arthritis; SpA: spondyloarthritis; MTX: methotrexate. Data represent number of patients in each category.

### Association of initial infliximab concentration and development of ATI

**Figure 2:**
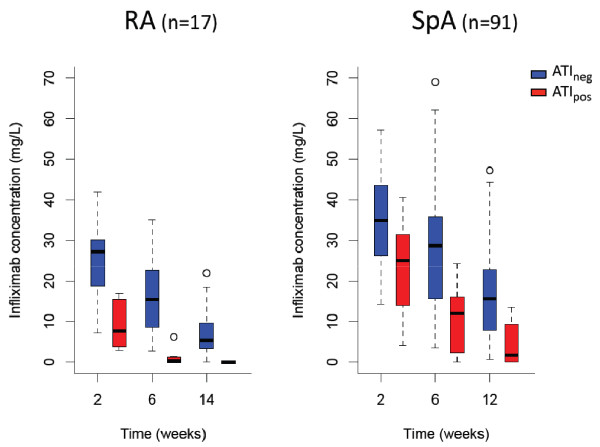
**Infliximab concentrations at initiation in ATI_pos _and ATI_neg _patients**. Box plots show the medians and interquartile ranges, and whisker plots represent the 95th percentiles. ATI: antibodies toward infliximab; ATI_pos_: ATI detected at least once during follow-up; ATI_neg_: ATI not detected; RA: rheumatoid arthritis; SpA: spondyloarthritis.

Trough serum infliximab concentration during treatment initiation (weeks 2 to 14) was lower for ATI_pos _patients than for ATI_neg _patients for both diseases, with the difference being significant as early as week 2 (Table 3 and Figure 2). For 8 of the 21 ATI_pos _patients, therapy was discontinued because of concomitant infection or surgery, and ATI developed after infliximab therapy was resumed.

**Table 3:** Trough infliximab concentration (mg/L) during infliximab initiation for ATI_pos _and ATI_neg _patients with RA and SpA^a^

	RA (*n *= 17)			SpA (*n *= 91)		
	
Time after infliximab initiation	ATI_pos _(*n *= 7)	ATI_neg _(*n *= 10)	*P *value	ATI_pos _(*n *= 14)	ATI_neg _(*n *= 77)	*P *value
Week 2	7.7 (2.8 to 16.9)	27.2 (7.1 to 41.9)	0.002	25.0 (4.0 to 40.7)	35.8 (14.3 to 57.2)	0.003
Week 6	0.3 (0 to 6.2)	15.4 (2.7 to 35.0)	0.001	11.9 (0 to 24.2)	29.5 (3.4 to 69.0)	< 0.001
Weeks 12 and 14	0 (0 to 0.03)	5.4 (0 to 21.9)	0.01	1.6 (0 to 13.5)	15.8 (0.7 to 47.3)	< 0.001

^a^ATI: antibodies toward infliximab; ATI_pos_: ATI detected at least once during follow-up; ATI_neg_: ATI not detected; RA: rheumatoid arthritis; SpA: spondyloarthritis. Results are given as medians (ranges).

### Association of ATI, infusion reactions and maintenance of infliximab

Among ATI_pos _patients, 11 (52%) had at least one infusion-related reaction, as compared with only 1 (1%) in the ATI_neg _group. The median interval between ATI detection and infusion-related reactions was 42 days (0 to 702 days). These infusion-related reactions were rashes, hyperthermia, chills, Quincke's oedema and tachycardia. Among the 11 ATI_pos _patients who had a reaction to infusion, 4 required intravenous corticosteroids and intravenous antihistamines, and 2 required only oral antihistamines. One patient developed Guillain-Barré syndrome that partially improved after polyvalent immunoglobulin treatment. In four patients, no treatment was given.

**Figure 3:**
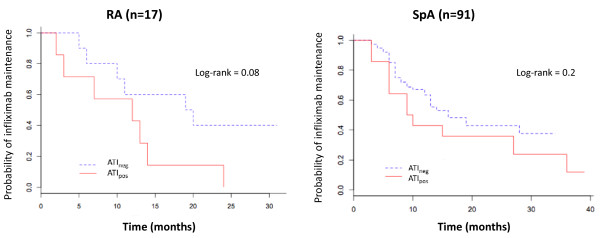
**Infliximab maintenance according to ATI status in RA and SpA patients**. ATI: antibodies toward infliximab; ATI_pos_: ATI detected at least once during follow-up; ATI_neg_: ATI not detected.

Eighteen (86%) of the ATI_pos _patients and forty-one (47%) of the ATI_neg _patients discontinued infliximab during follow-up. Events leading to treatment withdrawal significantly differed between the two groups (*P *< 0.001). In half of the 18 ATI_pos _patients, treatment was stopped because of infusion-related reactions, whereas in 31 (76%) of the 41 ATI_neg _patients treatment was stopped because of treatment failure (Table 4). Infliximab was maintained longer, but not significantly so, in ATI_neg _patients than in ATI_pos _patients for both diseases (Figure 3). ATI_neg _and ATI_pos _patients with RA showed maintenance of infliximab at a median of 19.5 months (5.0 to 31.0 months) and 12.0 months (2.0 to 24.0 months), respectively (*P *= 0.08). ATI_neg _and ATI_pos _patients with SpA showed maintenance of infliximab at a median of 16.0 months (3.0 to 34.0 months) and 9.5 months (3.0 to 39.0 months), respectively (*P *= 0.20).

**Table 4:** Causes of infliximab discontinuation in ATI_pos _and ATI_neg _patients^a^

Cause of discontinuation	ATI_pos _(*n *= 18)	ATI_neg _(*n *= 41)
Treatment failure		
Primary failure	2 (11%)	23 (56%)
Secondary failure	3 (17%)	8 (20%)
Infusion reactions	9 (50%)	1 (2%)
Adverse events	1 (5.5%)	6 (15%)
Other	1 (5.5%)	2 (5%)
Lost to follow-up	2 (11%)	1 (2%)

^a^ATI: antibodies toward infliximab; ATI_pos_: ATI detected at least once during follow-up; ATI_neg_: ATI not detected. All data are number of patients (%).

### Association of trough infliximab concentration after treatment initiation and maintenance of infliximab

**Figure 4:**
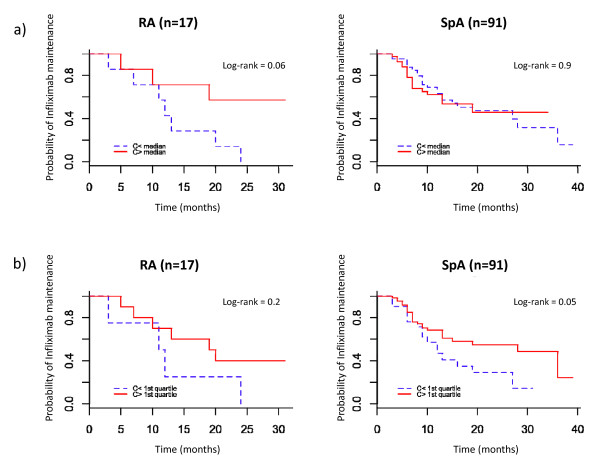
**Maintenance of treatment by trough infliximab concentration after treatment initiation**. RA patients were separated according to **(a) **the median and **(b) **the first quartile of trough infliximab concentrations measured at week 14. SpA patients were separated according to the **(a) **median and **(b) **the first quartile of trough infliximab concentrations measured at week 14. RA: rheumatoid arthritis; SpA: spondyloarthritis.

The association of maintenance of infliximab with infliximab concentration after treatment initiation is shown in Figure 4. RA patients whose trough infliximab concentration at week 14 was above the median (concentration > 3.2 mg/L) and above the first quartile (concentration > 0.05 mg/L) showed longer infliximab maintenance than other patients, although not significantly so (logrank = 0.06 and 0.2 respectively). For SpA patients whose trough concentration at week 12 was above the median (concentration > 13.7 mg/L), infliximab maintenance was no longer than that for other patients (logrank = 0.9). However, infliximab maintenance was longer for SpA patients with trough concentrations above the first quartile (concentration > 6.5 mg/L; logrank = 0.05) than for other patients.

## Discussion

Our study demonstrates that low trough infliximab concentration during treatment initiation is predictive of immunisation against infliximab on the basis of the presence of ATI. Previous studies have reported an association of low infliximab concentration 1.5 months after initiation and the development of ATI [4]. We found that more patients with than without ATI had a low infliximab concentration as early as two weeks after their first infusion. Furthermore, in some cases, the development of ATI occurred after a temporary discontinuation of infliximab. Under a certain threshold concentration of infliximab, during treatment initiation or even after a 'therapeutic holiday', patients may be at high risk of immunisation against infliximab. ATI are known to increase infliximab clearance, as previously reported in SpA and inflammatory bowel diseases, and could explain the nonresponse or loss of response in some cases [14,15]. Our findings argue for early and continuous monitoring of serum concentrations of mAb in drugs such as infliximab. Further studies are needed to support this hypothesis before it can be applied in clinical practice.

As previously reported, development of ATI is associated with poor maintenance of infliximab [5,6]. We found that ATI developed during follow-up in 41% of RA patients and 15% of SpA patients receiving infliximab. Our results are similar to those of previous studies reporting ATI in 43% of RA patients and 29% of SpA patients in the first year of treatment [5,6]. We found that immunisation against infliximab occurs early after treatment initiation, because we detected ATI in half of the ATI_pos _patients before a median of 3.7 months of treatment.

Immunogenicity of biopharmaceuticals is not restricted to chimeric mAb. In a large cohort study of RA patients treated with adalimumab, a fully human mAb also targeting TNFα, Bartelds *et al*. [16] reported that 76 (28%) of 272 patients developed antidrug antibodies (ADAs) after three years of treatment. In their study, ADAs were associated with a higher probability of treatment failure and drug discontinuation compared with ADA-negative patients. Immunisation appeared soon after treatment started: 67% of cases were detected during the first 28 weeks of therapy. The median time until detection of ATI in our study was 3.7 months, which is in good agreement with the results of the study by Bartelds *et al*. [16].

The development of ATI was also associated with increased risk of infusion reactions, as already reported [4-6]. In our experience, half of ATI_pos _patients experience such reactions, often soon after the detection of ATI, which leads to infliximab discontinuation.

Of note, because of drug intolerance, only 53% of our RA patients received MTX with infliximab. This situation may account for the rather high frequency of immunisation observed in our study. The role of MTX in preventing ATI formation in RA was suspected by Maini *et al*. [1] and Bendtzen *et al*. [4]. In our study, ATI developed less often, although not significantly so, in RA patients receiving infliximab and MTX than in those receiving infliximab alone, but the small number of patients (*n *= 17) prevents us from drawing any definite conclusions. To our knowledge, the present study provides the first evidence of a significant association of the use of MTX and a reduced risk of ATI development in SpA. Breban et *al*. [17] tested MTX in combination with infliximab in a subset of ankylosing spondylitis patients receiving treatment with infliximab by using an on-demand strategy. They showed a trend toward fewer reactions to infusions in the group receiving MTX as compared with the group not receiving MTX, although these results were not statistically significant. The fact that none of our SpA patients with MTX have developed ATI suggests that MTX is a credible factor in reducing immunisation and should be given in combination with mAb.

High trough infliximab concentrations measured at the end of treatment initiation (that is, before the fourth infusion) seem to predict infliximab maintenance in both RA and SpA. This result is in agreement with the findings of our previous reports and suggests that early monitoring and dosage adjustment of underexposed patients could improve long-term infliximab maintenance [18,19].

## Conclusion

In summary, almost 20% of patients with rheumatic diseases who received infliximab showed ATI during follow-up, half of them before four months after treatment initiation. High initial serum concentration of infliximab reduces the development of ATI, and absence of ATI seems to prolong maintenance of infliximab. Taken together, these findings argue for early monitoring of infliximab serum concentrations and should be confirmed in a prospective study.

## Abbreviations

ATI: antibody toward infliximab; BASDAI: Bath Ankylosing Spondylitis Disease Activity Index; CRP: C-reactive protein; ELISA: enzyme-linked immunosorbent assay; ESR: erythrocyte sedimentation rate; mAb: monoclonal antibody; MTX: methotrexate; RA: rheumatoid arthritis; SpA: spondyloarthritis; TNFα: tumour necrosis factor α.

## Competing interests

ED, DCML, FL, DT and HW have no competing interests to declare. DM received grant/research support from Abbott Laboratories. GP is a consultant for Laboratoires Français du Fractionnement et des Biotechnologies, Roche and Wyeth. PG received grant/research support from Abbott Laboratories, Schering-Plough, Wyeth, Roche and Bristol-Myers Squibb and is a consultant for Laboratoires Français du Fractionnement et des Biotechnologies, Roche, Schering-Plough and Wyeth.

## Authors' contributions

ED and DM drafted the manuscript, supervised the study design and performed the statistical analysis. GP and PG helped draft the manuscript. DT and HW supervised the immunoassays. DCML and FL performed clinical measurements. All authors approved the final manuscript.
